# Special Issue: “Advances in Osteogenesis”

**DOI:** 10.3390/ijms26199602

**Published:** 2025-10-01

**Authors:** Andrea Del Fattore

**Affiliations:** Bone Physiopathology Research Unit, Translational Pediatrics and Clinical Genetics Research Division, Bambino Gesù Children’s Hospital, IRCCS, 00146 Rome, Italy; andrea.delfattore@opbg.net

## Results

Alterations in bone formation activity lead to the onset of several bone diseases characterized by increased or reduced bone mass; the consequence of the unbalanced bone formation can be severe, and in some cases lethal [1]. Understanding the molecular and cellular mechanisms that control the osteogenesis process is therefore essential to dissect the signaling pathways involved in bone formation and identify new therapeutic targets for rare bone diseases. Bone formation is a highly coordinated process that involves the proliferation and differentiation of mesenchymal progenitor cells into osteoblasts, the synthesis and deposition of extracellular matrix proteins, and the mineralization of this matrix to ensure mechanical strength. Disruptions at any of these steps can result in pathological conditions that significantly affect skeletal health [2].

Moreover, osteogenesis plays a central role in bone healing following fractures, trauma, or surgical interventions [3]. However, in elderly patients, individuals with metabolic disorders, or those receiving long-term corticosteroid therapy, bone healing can be severely impaired. For this reason, there is a strong clinical interest in understanding how systemic factors such as hormones, cytokines, and mechanical loading influence the recruitment and differentiation of osteoprogenitor cells.

In recent years, significant progress has been made in identifying signaling molecules, growth factors, and transcriptional regulators that play key roles in osteoblast differentiation and bone matrix mineralization. Notably, pathways such as Wnt/β-catenin, BMP/SMAD, and Notch signaling have been recognized as critical determinants of osteogenic commitment [4]. Dysregulation of these pathways can result in conditions such as osteoporosis, high bone mass disease, and other skeletal dysplasias, which can profoundly impact patient quality of life and lead to a higher risk of fractures. Advances in genomics and single-cell analysis have been important to understand the heterogeneity of osteogenic cells and their response to mechanical, hormonal, and inflammatory stimuli [4].

In parallel, the development of novel biomaterials and scaffold technologies has opened new frontiers for tissue engineering and regenerative medicine. Three-dimensional (3D) bioprinting allows for the precise fabrication of bone-like constructs with controlled porosity and mechanical properties, providing a supportive microenvironment for osteoblasts and stem cells [5]. Combining these scaffolds with bioactive molecules such as bone morphogenetic proteins or vascular endothelial growth factor (VEGF) has been shown to enhance osteogenesis and angiogenesis, two processes that are closely linked during bone regeneration [5,6,7]. Moreover, gene-editing approaches such as CRISPR-Cas9 offer the possibility to correct pathogenic mutations or engineer cells with enhanced regenerative potential, paving the way for personalized therapies [8]. 

Beyond experimental approaches, computational models and artificial intelligence (AI) are increasingly being integrated into bone research. Machine learning algorithms can predict the risk of fractures, analyze imaging data to detect signs of bone degeneration, and simulate patient-specific responses to therapeutic treatments. These tools have the potential to transform clinical decision-making by enabling earlier diagnosis, more accurate prognosis, and tailored treatment plans [9,10].

Overall, the convergence of molecular biology, bioengineering, and computational science is accelerating the discovery of new therapeutic strategies for bone disorders. Continued interdisciplinary collaboration will be essential to translate these advance into clinical practice and provide better outcomes for patients affected by impaired bone mass, delayed fracture healing, or rare skeletal diseases.
